# D-Ribose Induces Podocyte NLRP3 Inflammasome Activation and Glomerular Injury via AGEs/RAGE Pathway

**DOI:** 10.3389/fcell.2019.00259

**Published:** 2019-10-30

**Authors:** Jinni Hong, Guangbi Li, Qinghua Zhang, Joseph Ritter, Weiwei Li, Pin-Lan Li

**Affiliations:** ^1^Integrated Laboratory of Traditional Chinese Medicine and Western Medicine, Peking University First Hospital, Beijing, China; ^2^Department of Gynecology, The Affiliated People’s Hospital of Fujian University of Traditional Chinese Medicine, Fuzhou, China; ^3^Department of Pharmacology and Toxicology, Medical College of Virginia, Virginia Commonwealth University, Richmond, VA, United States

**Keywords:** pentose monosaccharide, inflammatory machinery, AGEs-RAGE system, glomerular disease, end-stage renal disease

## Abstract

D-ribose levels are demonstrated to be increased in type II diabetes mellitus and increased blood D-ribose is involved in the development of diabetic complications such as diabetic encephalopathy and nephropathy. However, the mechanism mediating the pathogenic role of D-ribose in nephropathy remains poorly understood. Given that D-ribose was reported to induce advanced glycation end products (AGEs) formation, the present study tested whether D-ribose induces NLRP3 activation and associated glomerular injury via AGEs/receptor of AGEs (RAGE) signaling pathway. *In vivo*, C57BL/6J and Asc^–/–^ mice were treated with D-ribose with or without AGEs inhibitor. Administration of D-ribose daily for 30 days was found to induce NLRP3 inflammasome formation in glomerular podocyte, as shown by increased co-localization of NLRP3 with apoptosis-associated speck-like protein containing a caspase recruitment domain (ASC) or caspase-1. This D-ribose-induced NLRP3 inflammasome formation was accompanied by its activation as evidenced by increased IL-1β production, a major product of NLRP3 inflammasome. Corresponding to NLRP3 inflammasome activation, D-ribose led to significant glomerular injury in mice. All these D-ribose-induced glomerular inflammasome and associated pathological changes were markedly attenuated by deletion of Asc gene. Furthermore, the accumulation of AGEs and RAGE was found increased in glomeruli of mice receiving D-ribose. In cell studies, we also confirmed that D-ribose induced NLRP3 inflammasome formation and activation in podocytes, which was significantly blocked by caspase-1 inhibitor, YvAD. Mechanically, AGEs formation inhibition and cleavage or silencing of RAGE gene were shown to suppress D-ribose-induced NLRP3 inflammasome formation and activation, as shown by significant reduction of NLRP3 inflammasome molecular aggregation, caspase-1 activity and IL-1β production. These results strongly suggest that relatively long term administration of D-ribose induces NLRP3 inflammasome formation and activation in podocytes via AGEs/RAGE signaling pathway, which may be one of important triggering mechanisms leading to diabetic nephropathy.

## Introduction

D-ribose is a naturally occurring monosaccharide present in all living cells. It is mainly supplied in food and widely recommended as a supplement of metabolic therapy for chronic fatigue syndrome and coronary artery disease (Perlmutter et al., 1991; Teitelbaum et al., 2006). The beneficial effect of D-ribose is to lower malondialdehyde and glutathione levels during hypoxic stress (Gross et al., 1989; Seifert et al., 2009). However, a potential adverse effect of ribosylation was raised recently. The non-enzymatic glycation of proteins by reduction of D-ribose may lead to fructosamine formation (Day et al., 1979) and irreversible production of advanced glycation end products (AGEs), the toxic glycated proteins (Wei et al., 2009). Recently, D-ribose as a potential risk factor in the development of type II diabetes mellitus (T2DM) has been considered being overlooked (Su and He, 2014), given that D-ribose produces AGEs much more rapidly than D-glucose (Chen et al., 2009; Wei et al., 2009). Patients with diabetes mellitus (DM) suffering from metabolic derangements and organ complications may be associated not only with D-glucose but also with D-ribose (Babaei-Jadidi et al., 2003; Wei et al., 2009, 2012; Lopez-Clavijo et al., 2014; Tessier et al., 2016). It has been reported that in T2DM patients, blood and urine D-ribose levels were increased, which may trigger tissue inflammation, Tau protein hyper-phosphorylation, and amyloid β-like deposits in brain, resulting in cognitive impairment (Han et al., 2011, 2014; Wu et al., 2015). In a recent study, we also found that D-ribose induced glomerular inflammation and sclerosis with increased AGEs level in mouse kidney. However, it remains unknown how D-ribose induces glomerular inflammation and leads to glomerular injury.

Accumulating evidence shows that the formation and activation of Nod-like receptor protein 3 (NLRP3) inflammasome in podocytes may instigate glomerular inflammation and lead to ultimate glomerular sclerosis in response to pathological stimuli such as hyperhomocysteinemia (hHcy), obesity and DM (Yi et al., 2006; Abais et al., 2013, 2014a). The NLRP3 inflammasome serves as an intracellular inflammatory machinery, which can be activated by a wide range of danger signals and lead to various chronic degenerative diseases (Zhang et al., 2012; Yin et al., 2013; Bakker et al., 2014). This novel cytosolic multiprotein complex is composed of NLRP3, the adaptor molecule apoptosis-associated speck-like protein containing a CARD (caspase recruitment domain) (ASC), and the cysteine protease caspase-1. During formation and activation of the NLRP3 inflammasome, cleaved caspase-1 causes the maturation of pro-inflammatory cytokines, such as IL-1β and IL-18. As a canonical mechanism, secretion of these inflammatory cytokines to extracellular space triggers tissue inflammatory response (Martinon and Tschopp, 2005). Furthermore, NLRP3 inflammasome activation may also induce cell metabolic disturbance, cell transformation, and tissue damage as its uncanonical effects, which may be another important triggering mechanism for organ diseases beyond inflammation (Yang et al., 2014). To our knowledge, so far there is no study that links the detrimental effects of D-ribose to NLRP3 inflammasome activation in glomerular injury or sclerosis.

The present study was designed to test the hypothesis that D-ribose induces NLRP3 inflammasome formation and activation in podocytes in an AGEs dependent manner, which will be an important triggering mechanism leading to podocyte injury and glomerular sclerosis. We first demonstrated that D-ribose induced podocyte injury and glomerular sclerosis in wide-type (WT) mice compared with Asc gene knockout mice. We also observed whether the pathological role of D-ribose to induce NLRP3 inflammasome activation is associated with increased production of AGEs. Then, we performed *in vitro* studies to test whether D-ribose-induced NLRP3 inflammasome activation is associated with AGEs-RAGE signaling pathway. These studies together confirmed the key role of NLRP3 inflammasome activation in D-ribose-induced podocyte injury and consequent glomerular sclerosis, which is mediated by AGEs-RAGE signaling pathway.

## Materials and Methods

### Animals

Eight-week-old, male C57BL/6J (The Jackson Laboratory, Bar Harbor, ME, United States) were intraperitoneally (i.p.) injected vehicle or D-ribose (dissolved in 0.9% saline) at a dose of 2 g/kg BW, once a day, for 30 days. In another series, male ASC^–/–^ mice and their wild-type littermates at the same age were used for confirmation of NLRP3 inflammasome involvement in the action of D-ribose. All mice were randomly distributed to Vehl (Vehicle), D-R (D-ribose) and D-R + AG (D-ribose + aminoguanidine, AGEs formation inhibitor) groups, 8 mice in each group. Mice of D−R + AG group were additionally fed with 1 g/L AG in water for 30 days (Yavuz et al., 2001). All mice were housed under identical conditions in a pathogen-free environment with a 12:12 h light/dark cycle and free access to laboratory chow and water. Mice were acclimatized to the housing environment for at least 1 week before the experiments. 3 days before the protocol was finished, mice were placed in metabolic cages to collect urine samples for analysis of urinary albumin and protein excretion. On the day protocol was completed, blood samples were taken for measurement of fasting blood glucose with OneTouch Ultra2 blood glucose meter (LifeScan Europe, Switzerland). Then, mice were sacrificed under mild ethyl ether anesthesia and their kidneys were harvested. All animal experimental protocols were approved by the Institutional Animal Care and Use Committee of the Virginia Commonwealth University.

### Cell Culture

A conditionally immortalized mouse podocyte cell line (Graciously provided by Dr. P. E. Klotman, Division of Nephrology, Department of Medicine, Mount Sinai School of medicine, New York, NY, United States), were cultured and maintained as described before (Abais et al., 2013; Hong et al., 2019). For all experiments, culture medium was replaced with serum-free medium for 24 h prior to treatments. Podocytes were incubated with 25 mM D-ribose (Sigma, United States), 25 mM L-ribose (AK scientific, United States) as negative control (AK scientific, United States) and 25 mM D-glucose (Sigma, United States) as positive control for 24 h. To inhibit caspase-1 activity in podocytes, its selective inhibitor, Ac-YVAD-CMK (YvAD, 10 μg/ml, Cayman Chemical) was used 30 min prior to treatments. To inhibit the role of AGEs, AGEs formation inhibitor aminoguanidine (AG, 50 μM, Sigma Aldrich) and a breaker of AGEs-based cross-links, alagebrium chloride (ALT, 100 μM, TCI AMERICA) were used 30 min prior to treatments (Dhar et al., 2016; Chowdhury, 2017).

### Glomerular Morphological Examinations

Kidneys were fixed with 4% (v/v) paraformaldehyde (PFA) in PBS, embedded with paraffin, sliced into 4 μm sections and stained with Periodic Acid-Schiff. Glomerular morphology was observed and assessed semi-quantitatively as described previously (Raij et al., 1984; Abais et al., 2014b).

### Urinary Protein and Albumin Measurements

Total urinary protein concentrations were determined spectrophotometrically using Bradford assay (Sigma, United States). Urinary albumin concentration was measured with mouse albumin ELISA kit (Bethyl Laboratories, Montgomery, TX, United States) according to manufacturer’s instructions.

### Immunohistochemistry

After embedded and sectioned, slides were incubated with primary antibody against IL-1β (1:200, R&D Systems, United States), RAGE (1:200, Sigma, United States) and AGEs (1:200, Abcam, Cambridge, MA, United States) at 4°C overnight. Then slides were incubated with biotinylated secondary antibodies and a streptavidin peroxidase complex (PK-7800, Vector Laboratories, Burlingame, CA, United States). Finally, samples observed with microscopy as described previously (Raij et al., 1984; Hong et al., 2019). The area percentage of the positive staining was calculated with Image Pro Plus 6.0 software (Raij et al., 1984).

### Immunofluorescence Microscopy

After treatments, kidney slides and podocyte culture coverslips were fixed, blocked and incubated with primary antibodies against NLRP3 (1:100, Abcam, Cambridge, MA, United States), ASC (1:200, Santa Cruz Biotechnology, Dallas, TX, United States), cleaved-caspase-1 (1:200, Santa Cruz Biotechnology, Dallas, TX, United States), podocin (1:400, Sigma), or desmin (1:400, Thermo Fisher Scientific) at 4°C overnight. Then slides were incubated with corresponding second antibodies with either Alexa- 488- or Alexa-555-labeled (Invitrogen, Carlsbad, CA, United States). For example, slides incubated with NLRP3 were then incubated with donkey anti goat secondary antibody, Alexa fluor plus 488, slides incubated with ASC, cleaved-caspase-1 or Podocin were then incubated with donkey anti mouse secondary antibody, Alexa fluor plus 555, slides incubated with desmin were then incubated with donkey anti rabbit secondary antibody, Alexa fluor plus 488. After that, slides were observed with a laser scanning confocal microscope (Fluoview FV1000, Olympus, Japan). Co-localization coefficient was analyzed with Image Pro Plus 6.0 software and presented by Pearson’s correlation coefficient (PCC).

### Western Blot Analysis

Equivalent amount of proteins (20–30 μg) was resolved on SDS-PAGE gels and transferred to PVDF membrane. After blocking, membranes were probed with primary antibodies rabbit anti-Cle-Caspase-1 (1:1000, cell signaling technology), rabbit anti-pro-Caspase-1 (1:1000, Abcam, Cambridge, MA) and rabbit anti-β-actin (1:10000, Santa Cruz Biotechnology, Dallas, TX, United States) at 4°C overnight. After incubated with donkey anti-rabbit-HRP IgG (1:5000, Santa Cruz Biotechnology, Dallas, TX, United States), immunoreactive bands were detected by chemiluminescence techniques with LI-COR Odyssey Fc. The intensity of the specific bands was calculated with ImageJ software (NIH, Bethesda, MD, United States).

### Assays of Caspase-1 Activity and IL-1β Production

Caspase-1 activity was measured with a commercial colorimetric assay kit (Biovision, Mountain View, CA, United States), with OD = 405 nm, and IL-1β production in cell supernatant was detected with mouse IL-1β ELISA kit (R&D systems, United States), with OD = 450 nm, according to manufacturer’s instructions.

### RNA Interference of RAGE

RAGE small interference RNAs (siRNAs) was purchased from Santa Cruz Biotechnology, Dallas, TX, United States. SiRNAs transfection was performed with the silent Lipid Reagent (Bio-Rad, United States) according to manufacturer’s instructions.

### Statistical Analysis

Data are presented as means ± SE. The significant differences between and within multiple groups were examined using one way or two-way ANOVA, followed by Duncan’s multiple-range test. *P* < 0.05 was considered statistically significant.

## Results

### D-Ribose Induced Glomerular Dysfunction and Injury in an AGEs-Dependent Manner

**FIGURE 1:**
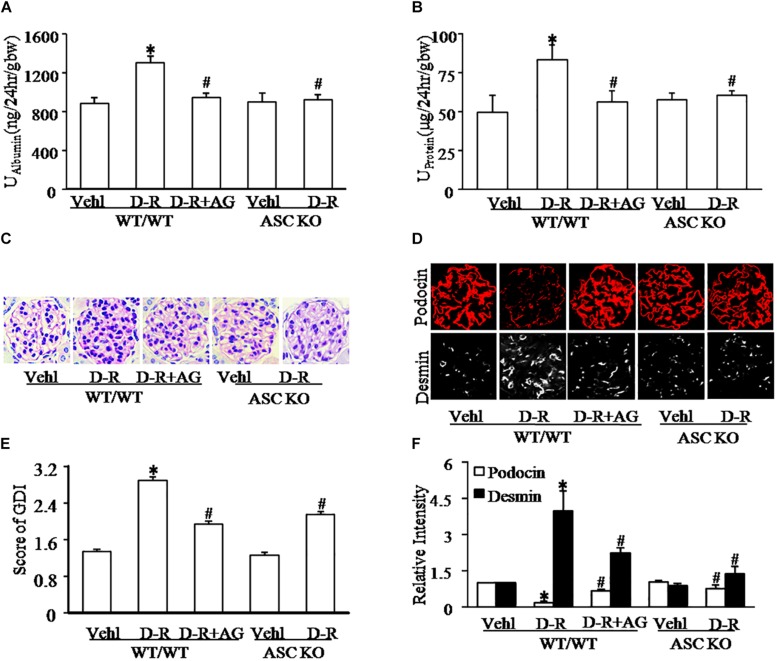
D-ribose induced glomerular dysfunction and injury in an AGEs-dependent manner. **(A)** Urine albumin excretion of mice with different genotypes and treatment (*n* = 6). **(B)** Urine protein excretion of mice with different genotypes and treatment (*n* = 6). **(C)** Representative images of glomerular morphological changes (Periodic Acid-Schiff staining) from mice with different genotypes and treatments (*n* = 5). **(D)** Representative images of immunostained glomeruli for podocin and desmin from mice with different genotypes and treatments (*n* = 5). **(E)** Summarized data showing glomerular damage index (GDI) (Periodic Acid-Schiff staining) from mice with different genotypes and treatments (*n* = 5). **(F)** Summarized data of immunostained glomeruli for podocin and desmin from mice with different genotypes and treatments (*n* = 5). ^∗^*P* < 0.05 versus WT/WT-Vehl group, ^#^*P* < 0.05 versus WT/WT-D-R group. Vehl, Vehicle; D-R, D-ribose; AG, Aminoguanidine.

We first tested whether D-ribose treatment induced podocyte injury and glomerular damage in WT and Asc gene knockout mice. As shown in Figures 1A,B, i.p. injection of D-ribose for 30 days induced proteinuria and albuminuria in WT mice in comparison with mice treated with vehicle, which were markedly blocked by simultaneous administration of AG, an AGEs formation inhibitor. In Asc gene knockout mice, D-ribose-induced proteinuria and albuminuria were much less severe. Consistently, WT mice receiving D-ribose injection had remarkable extracellular matrix and collagen deposition, mesangial cell expansion and capillary collapse, indicating a typical glomerular sclerotic pathology (Figures 1C,E), which were abolished by AG co-treatment. Using confocal microscopy, D-ribose was found to markedly alleviate podocin staining, but increase the fluorescence intensity of desmin staining (Figures 1D,F) in glomeruli of WT mice. After injection of D-ribose for 30 days, fasting-blood glucose in D-ribose-treated mice was significantly decreased compared to Vehl group (128.33 mg/dL vs. 161.83 mg/mL). All these glomerular and podocyte injuries induced by D-ribose were substantially blocked by administration of AG. Additionally, in Asc gene knockout mice, D-ribose treatment failed to induce podocyte injury and glomerular damage, as shown by no differences in all measured glomerular injurious parameters between mice receiving Vehl and D-ribose treatments.

### Attenuation of D-Ribose-Induced NLRP3 Inflammasome Activation by AG in Glomeruli

**FIGURE 2:**
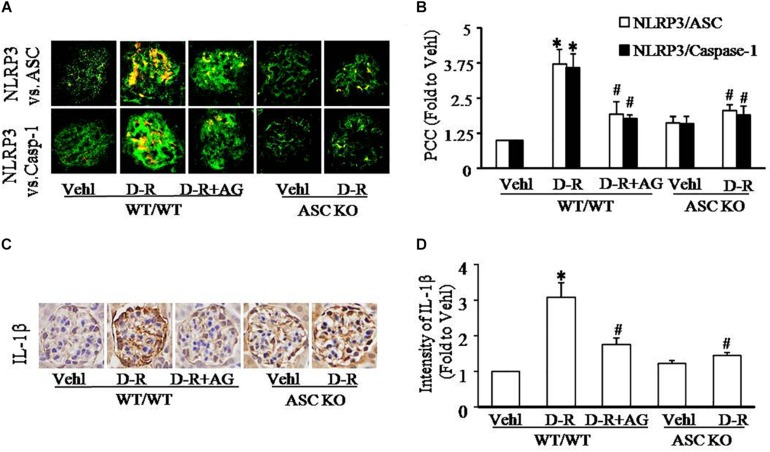
D-ribose-induced NLRP3 inflammasome formation and activation in glomeruli. **(A)** Representative confocal microscopic images showing co-localization of NLRP3 (green) with ASC (red) or with caspase-1 (red) from mice with different genotypes and treatments (*n* = 5). **(B)** Summarized data showing co-localization of NLRP3 (green) with ASC (red) or with caspase-1(red) in glomeruli from mice with different genotypes and treatment (*n* = 5). **(C)** Representative photomicrographs depicting immunohistochemical staining of glomeruli with anti-IL-1β antibody from mice with different genotypes and treatments (*n* = 5). **(D)** Summarized data of immunohistochemical staining of glomeruli for IL-1β level from mice with different genotypes and treatments (*n* = 5). ^∗^*P* < 0.05 versus WT/WT-Vehl group, ^#^*P* < 0.05 versus WT/WT-D-R group. Vehl, Vehicle; D-R, D-ribose; AG, Aminoguanidine.

As depicted in Figure 2A, the confocal microscopic analysis demonstrated that D-ribose remarkably increased co-localization of NLRP3 with ASC or caspase-1 (increased yellow staining) in glomeruli compared with Vehl group, which was blocked by AG treatment. Quantitation of the NLRP3 co-localization shows that NLRP3 inflammasome formation only occurred in mice receiving D-ribose, but not in mice of Vehl group (Figure 2B). Based on our previous studies showing that NLRP3 molecules are most enriched in podocytes, these results indicate the formation of NLRP3 inflammasome in glomerular podocytes of mice receiving D-ribose. Consistently, IL-1β as a prototype inflammatory cytokine produced by the NLRP3 inflammasome also significantly increased in glomeruli of mice treated with D-ribose compared to mice in Vehl group, which was markedly attenuated by AG (Figures 2C,D). In Asc gene knockout mice, however, D-ribose failed to induce NLRP3 inflammasome formation and activation in glomeruli. These findings demonstrate that NLRP3 inflammasome activation and consequent IL-1β production may contribute to D-ribose-induced podocyte injury and glomerular sclerosis.

### Enhancement of AGEs Production and RAGE Expression in Glomeruli of D-Ribose-Treated Mice

**FIGURE 3:**
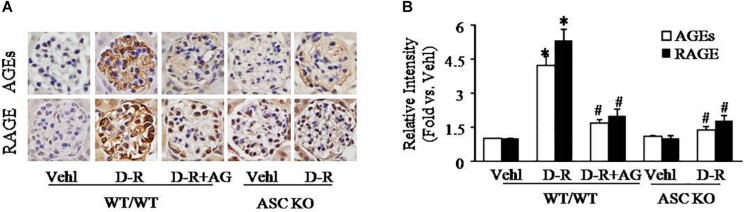
Enhancement of AGEs production and RAGE expression in glomeruli of D-ribose-treated mice. **(A)** Representative photomicrograph showing immunohistochemical staining of glomeruli with anti-AGEs and RAGE antibodies in mice with different genotypes and treatments (*n* = 5). **(B)** Summarized data of immunohistochemical staining of glomeruli for AGEs and RAGE level from mice with different genotypes and treatments (*n* = 5). ^∗^*P* < 0.05 versus WT/WT-Vehl group, ^#^*P* < 0.05 versus WT/WT-D-R group. Vehl, Vehicle; D-R, D-ribose; AG, Aminoguanidine.

To explore the mechanisms by which D-ribose induces NLRP3 inflammasome activation and podocyte injury, we observed changes in AGEs formation and RAGE expression in mouse glomeruli. By immunohistochemistry, AGEs and RAGE were remarkably elevated in glomeruli of mice receiving D-ribose compared to Vehl group, and it could be totally blocked by AG, the AGEs formation inhibitor (Figures 3A,B).

### Effects of D-Ribose on NLRP3 Inflammasome Formation and Activation in Podocytes

**FIGURE 4:**
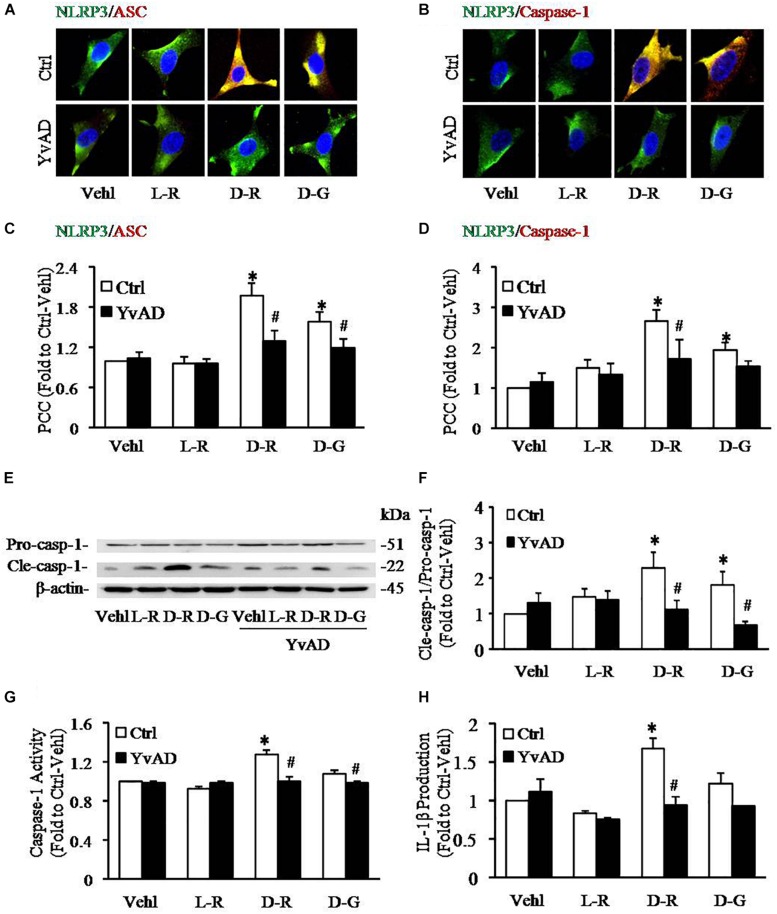
Effects of D-ribose on NLRP3 inflammasome formation and activation in podocytes. **(A)** Representative confocal microscopic images showing co-localization of NLRP3 (green) with ASC (red) (*n* = 6). **(B)** Representative confocal microscopic images showing co-localization of NLRP3 (green) with caspase-1 (red) (*n* = 6). **(C)** Summarized data depicting co-localization of NLRP3 (green) with ASC (red) during different treatments of podocytes (*n* = 6). **(D)** Summarized data depicting co-localization of NLRP3 (green) with caspase-1 (red) during different treatments of podocytes (*n* = 6). **(E)** Representative Western blot gel document of pro-Caspase-1 and Cle-Caspase-1 in podocytes with different treatments (*n* = 5). **(F)** Summarized data of pro-Caspase-1 and Cle-Caspase-1 in podocytes with different treatments (*n* = 5). **(G)** Caspase-1 activity in podocytes with different treatments (*n* = 6). **(H)** IL-1β levels in the supernatant of podocyte cultures with different treatments (*n* = 5). ^∗^*P* < 0.05 versus Ctrl-Vehl group, ^#^*P* < 0.05 versus Ctrl group. Ctrl, control; Vehl, Vehicle; L-R, L-ribose; D-R, D-ribose; D-G, D-glucose.

To further explore the mechanisms by which D-ribose activates NLRP3 inflammasome, we performed *in vitro* experiments in cultured podocytes. Caspase-1 inhibitor, YvAD, was used to pre-treat podocytes to test whether D-ribose indeed activates the NLRP3 inflammasome in podocytes in comparison with its isoform, L-ribose and positive control, D-glucose. By using confocal microscopy, we demonstrated that co-localization of NLRP3 (green) with ASC (red) or caspase-1 (red), an indicative of NLRP3 inflammasome formation, was much higher in podocytes treated with D-ribose than Vehl and L-ribose, but similar to podocytes treated with D-glucose. However, prior treatment of podocytes with YvAD almost completely abolished the enhanced co-localization of NLRP3 with ASC or caspase-1 induced by D-ribose or D-glucose (Figures 4A,B). The co-localization coefficient was summarized and is shown below the representative confocal microscopic images (Figures 4C,D).

Based on previous studies, increased cleavage of caspase-1 and enhanced caspase-1 activity and consequent IL-1β production reflect NLRP3 inflammasome activation. The present study found that D-ribose treatment remarkably increased the level of cleaved caspase-1 (22 kDa), but not procaspase-1(51 kDa) (Figures 4E,F), caspase-1 activity (Figure 4G) and IL-1β production (Figure 4H), and prior treatment with YvAD almost completely attenuated these changes. Additionally, we demonstrated that D-glucose had D-ribose-like effect on NLRP3 inflammasome activation in podocytes.

### Role of AGEs in D-Ribose-Induced NLRP3 Inflammasome Formation and Activation in Podocytes

**FIGURE 5:**
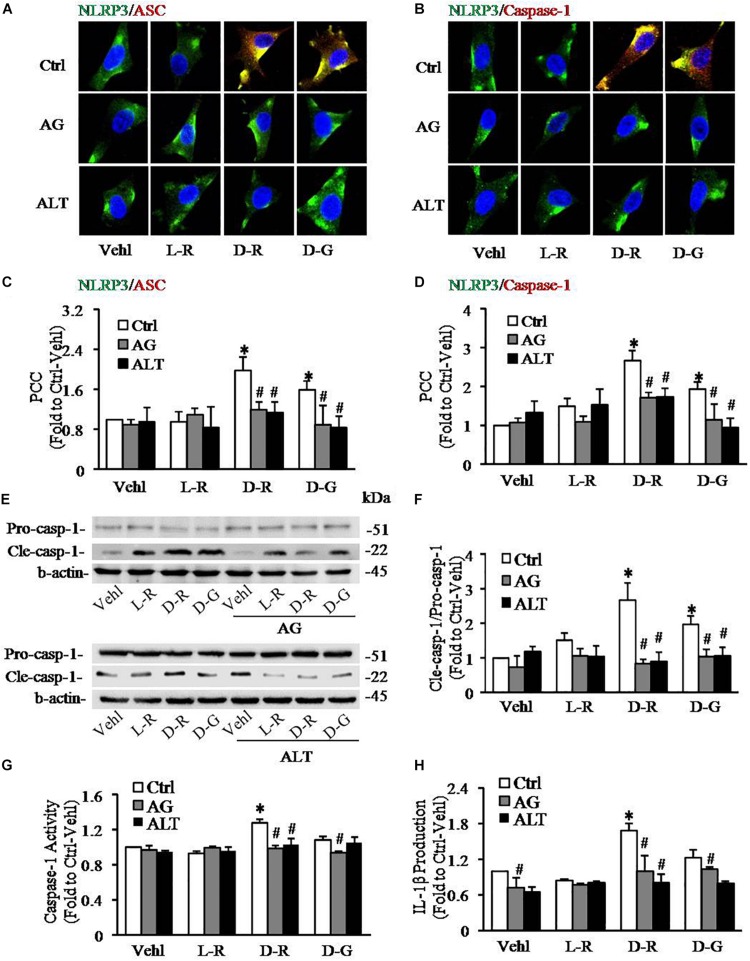
Effects of AGEs formation inhibition and their crosslink breakers on D-ribose-induced NLRP3 inflammasome formation and activation in podocytes. **(A)** Representative confocal microscopic images showing co-localization of NLRP3 (green) with ASC (red) (*n* = 6). **(B)** Representative confocal microscopic images showing co-localization of NLRP3 (green) with caspase-1 (red) (*n* = 6). **(C)** Summarized data depicting co-localization of NLRP3 (green) with ASC (red) during different treatments of podocytes (*n* = 6). **(D)** Summarized data depicting co-localization of NLRP3 (green) with caspase-1 (red) during different treatments of podocytes (*n* = 6). **(E)** Representative Western blot gel document of pro- Caspase-1 and Cle-Caspase-1 in podocytes with different treatments (*n* = 5–6). **(F)** Summarized data of pro-Caspase-1 and Cle-Caspase-1 in podocytes with different treatments (*n* = 5–6). **(G)** Caspase-1 activity in podocytes with different treatments (*n* = 6). **(H)** IL-1β levels in the supernatant of podocyte cultures with different treatments (*n* = 5). ^∗^*P* < 0.05 versus Ctrl-Vehl group, ^#^*P* < 0.05 versus Ctrl group. Ctrl, control; Vehl, Vehicle; L-R, L-ribose; D-R, D-ribose; D-G, D-glucose. AG, aminoguanidine. ALT, alagebrium chloride.

**FIGURE 6:**
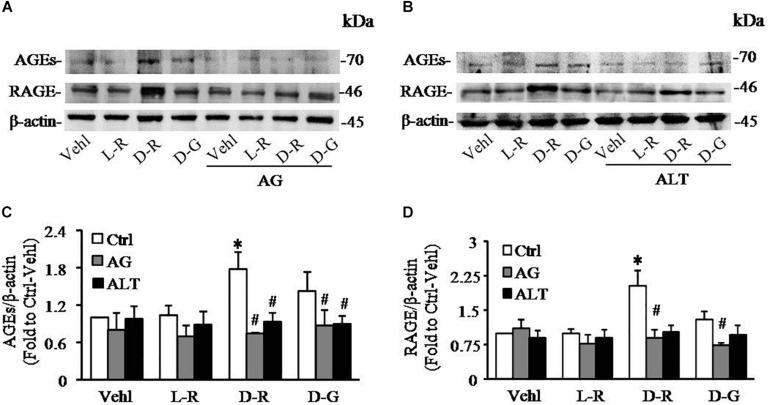
AG and ALT blocked D-ribose-induced AGEs production and RAGE overexpression in podocytes. **(A)** Representative western blot gel document of AGEs and RAGE in podocytes with different treatments (*n* = 6). **(B)** Representative western blot gel document of AGEs and RAGE in podocytes with different treatments (*n* = 6). **(C)** Summarized data of AGEs in podocytes with different treatments (*n* = 6). **(D)** Summarized data of RAGE in podocytes with different treatments (*n* = 6). ^∗^*P* < 0.05 versus Ctrl-Vehl group, #*P* < 0.05 versus Ctrl group. Ctrl, control; Vehl, Vehicle; L-R, L-ribose; D-R, D-ribose; D-G, D-glucose. AG, aminoguanidine. ALT, alagebrium chloride.

To further test whether AGEs play an important role in the formation and activation of NLRP3 inflammasome induced by D-ribose, AG, an AGEs formation inhibitor and alagebrium chloride (ALT), an AGEs breaker were used prior to incubation with D-ribose or vehicle. As shown in Figure 5, co-localization of NLRP3 with ASC (Figures 5A,C) or caspase-1 (Figures 5B,D) was much higher in D-ribose-treated podocytes than Vehl- or L-ribose treated podocytes, but similar to D-glucose-treated podocytes. However, prior treatment of podocytes with AG or ALT completely blocked D-ribose or D-glucose-induced co-localization of NLRP3 with ASC or caspase-1. The level of cleaved caspase-1 (Figures 5E,F), its activity (Figure 5G) and IL-1β production (Figure 5H) were remarkably elevated by D-ribose treatment, which was completely blocked by prior treatment of podocytes with AG or ALT. AG or ALT was also found to attenuate D-ribose-induced increased expression of AGEs and RAGE in podocytes (Figures 6A–D). D-glucose had a similar effect to D-ribose on NLRP3 inflammasome activation in podocytes, which was blocked by prior treatment of AG or ALT.

### RNA Interference of RAGE Prevented D-Ribose-Induced NLRP3 Inflammasome Formation and Activation in Podocytes

**FIGURE 7:**
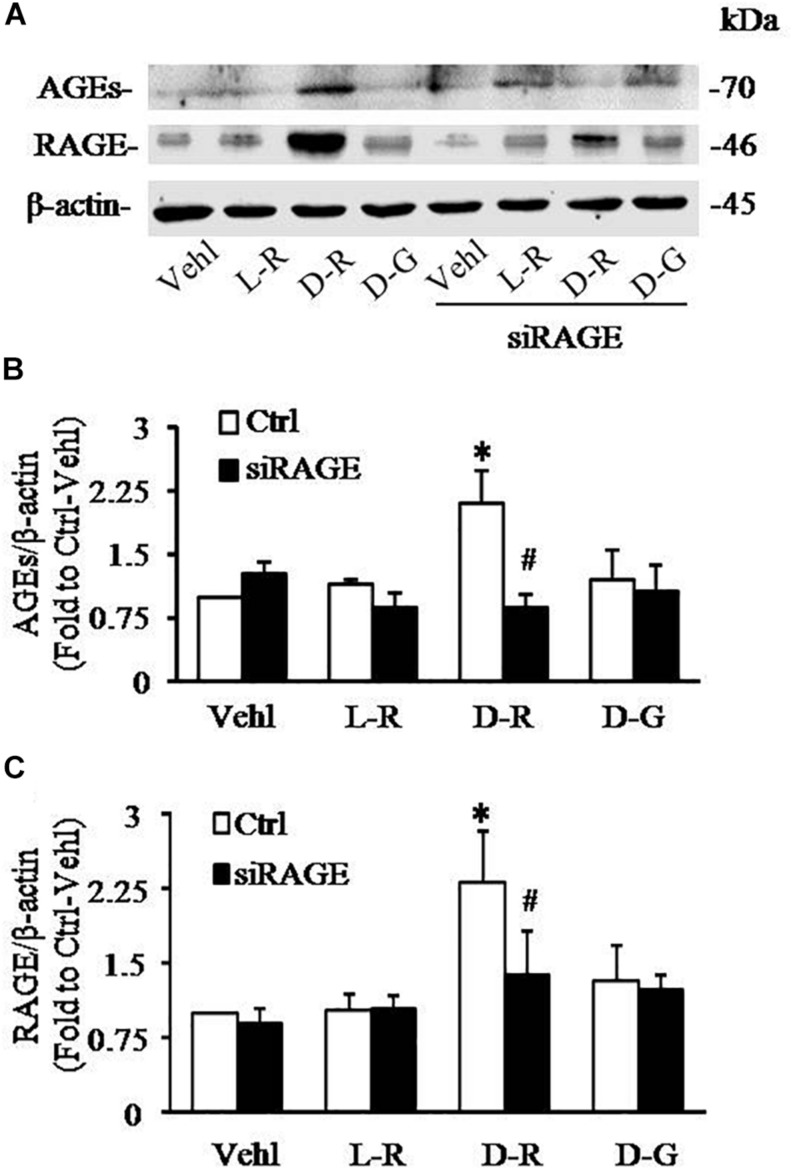
RNA interference of RAGE prevented D-ribose-induced NLRP3 inflammasome formation and activation in podocytes. **(A)** Representative western blot gel document of AGEs and RAGE in podocytes with different treatments (*n* = 6). **(B)** Summarized data of AGEs in podocytes with different treatments (*n* = 6). **(C)** Summarized data of RAGE in podocytes with different treatments (*n* = 6). ^∗^*P* < 0.05 versus Ctrl-Vehl group, ^#^*P* < 0.05 versus Ctrl group. Ctrl, control; Vehl, Vehicle; L-R, L-ribose; D-R, D-ribose; D-G, D-glucose.

**FIGURE 8:**
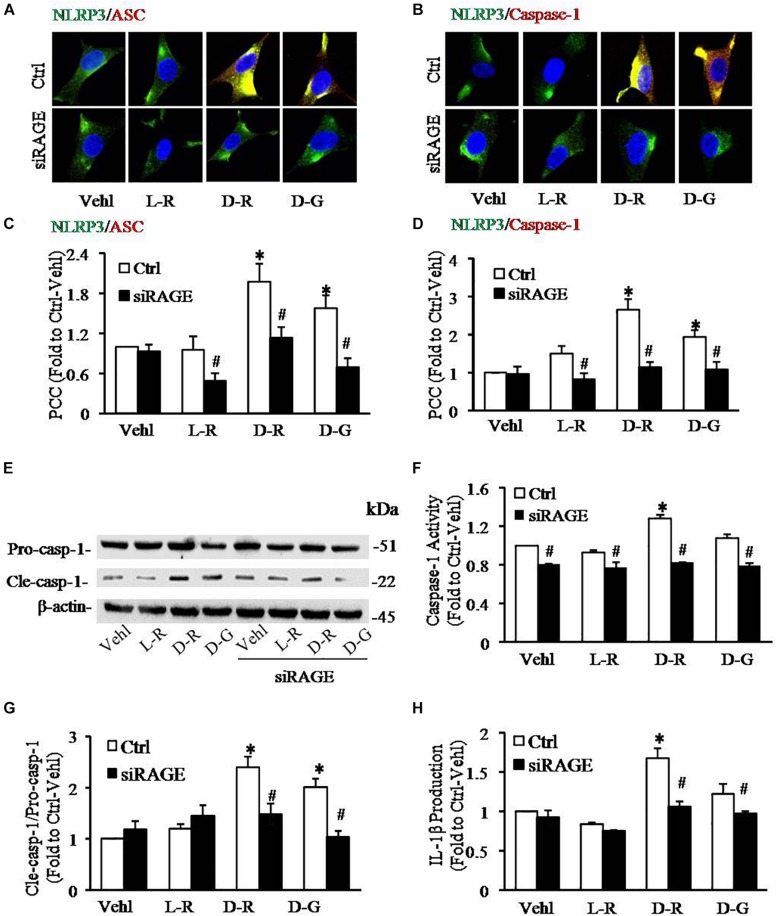
RNA interference of RAGE prevented D-ribose-induced NLRP3 inflammasome formation and activation in podocytes. **(A)** Representative confocal microscopic images showing co-localization of NLRP3 (green) with ASC (red) (*n* = 6). **(B)** Representative confocal microscopic images showing co-localization of NLRP3 (green) with caspase-1 (red) (*n* = 6). **(C)** Summarized data depicting co-localization of NLRP3 (green) with ASC (red) during different treatments of podocytes (*n* = 6). **(D)** Summarized data depicting co-localization of NLRP3 (green) with caspase-1 (red) during different treatments of podocytes (*n* = 6). **(E)** Representative Western blot gel document of pro-Caspase-1 and Cle-Caspase-1 in podocytes with different treatments (*n* = 5). **(F)** Caspase-1 activity in podocytes with different treatments (*n* = 5). **(G)** Summarized data of pro-Caspase-1 and Cle-Caspase-1 in podocytes with different treatments (*n* = 5). **(H)** IL-1β levels in the supernatant of podocyte cultures with different treatments (*n* = 5).^∗^*P* < 0.05 versus Ctrl-Vehl group, ^#^*P* < 0.05 versus Ctrl group. Ctrl, control; Vehl, Vehicle; L-R, L-ribose; D-R, D-ribose; D-G, D-glucose.

We further examined the role of RAGE in D-ribose-induced NLRP3 inflammasome formation and activation. In these experiments, a siRNA against RAGE (siRAGE) was transfected to podocytes prior to administration of D-ribose or controls. This siRNA effectively silenced RAGE expression and blocked D-ribose-induced increase in RAGE expression as shown by Western blot analysis (Figures 7A–C). By confocal microscopy, co-localization of NLRP3 (green) with ASC (red) or caspase-1 (red) was demonstrated to be much higher in D-ribose-treated podocytes than Vehl- or L-ribose-treated podocytes. RAGE gene silencing completely blocked D-ribose-induced co-localization of NLRP3 with ASC or caspase-1 (Figures 8A–D). RAGE gene silencing markedly diminished D-ribose-induced increase in cleaved caspase-1 levels (Figures 8E,G), caspase-1 activity (Figure 8F) and IL-1β production (Figure 8H). Similarly, D-glucose-induced NLRP3 inflammasome formation and activation in podocytes were also attenuated by RAGE gene silencing.

## Discussion

The present study was designed to determine whether D-ribose induces NLRP3 inflammasome activation and results in podocyte and glomerular injuries and whether it depends upon AGEs-RAGE signaling pathway. It was found that D-ribose induced NLRP3 inflammasome formation and activation, which led to podocyte injury and glomerular sclerosis in WT mice. Knockout of gene encoding Asc blocked D-ribose-induced NLRP3 inflammasome activation and consequent podocyte injury in mouse glomeruli. Furthermore, *in vitro* studies using cultured podocytes showed that D-ribose indeed stimulated NRLP3 inflammasome formation and activation. Inhibition of AGEs formation, increases in AGEs cleavage or silencing of RAGE gene all prevented D-ribose-induced NLRP3 inflammasome formation and activation, which protected podocytes from D-ribose-induced injury. These findings suggest that activation of RAGE by AGEs plays a crucial role in NLRP3 inflammasome activation and podocyte injury induced by D-ribose.

Diabetes mellitus is characterized by high blood glucose levels due to either insulin deficiency or insulin resistance, which generates many organ damages leading to degenerative complications such as diabetic retinopathy, and renal glomerular injuries. Although increased blood D-glucose level is widely known to be responsible for the development of diabetic complications, D-ribose is now emerging as a novel pathogenic factor for organ damages during DM. However, the molecular mechanism mediating the pathogenic action of D-ribose remains poorly understood. In the present study, we first demonstrated that i.p. injection of D-ribose induced podocyte injury and glomerular sclerosis in mice, which were associated with NLRP3 inflammasome activation and consequent increase in IL-1β production, suggesting that NLRP3 inflammasome activation may be a critical mechanism for D-ribose-induced podocyte injury and progressive development of glomerular sclerosis. In Asc gene knockout mice, D-ribose failed to produce NLRP3 inflammasome activation, which prevented D-ribose-induced podocyte injury and glomerular sclerosis. This further confirms that NLRP3 inflammasome activation is crucial for the pathogenic action of D-ribose. To our knowledge, these findings provide the first experimental evidence that D-ribose may induce podocyte injury and glomerular sclerosis via activation of the NLRP3 inflammasome. Recently, it has been reported that NLRP3 inflammasome activation serves as a triggering mechanism leading to glomerular injury and ultimate end-stage renal disease (ESRD) under different pathological conditions (Yi et al., 2006; Abais et al., 2014a). In this regard, there is evidence that the expression of NLRP3 inflammasome molecules and pro-inflammatory cytokines increased in patients and mice suffering from T2DM (Vandanmagsar et al., 2011; Lee et al., 2013). NLRP3 or caspase-1 gene knockout and caspase-1 inhibition blocked or even reversed the progression diabetic nephropathy in mice (Wen et al., 2011). All these results suggest that the NLRP3 inflammasome may be an important pathogenic factor for the development of diabetic nephropathy, which is probably a new target for treatment of DN complications in the kidney (Segelmark and Hellmark, 2010). Given that NLRP3 inflammasome activation may be initiated by the production of mitochondrial reactive oxygen species (ROS) and increased NADPH oxidase activity during DM (Gao et al., 2015), the action of D-ribose to activate NLRP3 inflammasome may be associated with this redox regulation because it can react with β_2_-microglobulin and induces the ribosylated protein through a ROS-mediated pathway (Kong et al., 2011).

As a reducing saccharide, D-ribose reacts with amino groups to initiate protein glycation and leads to the formation of AGEs, a type of major diabetic detrimental mixtures (Han et al., 2011). Previous studies showed that D-ribose induced AGEs accumulation (Han et al., 2011, 2014; Wei et al., 2012) and resulted in impairment of renal function (Wendt et al., 2003; Yeh et al., 2017). D-ribose is a stronger reducing sugar than D-glucose and it produces AGEs much more rapidly than D-glucose (Chen et al., 2009). In previous studies, the interaction between AGEs and RAGE has been confirmed to activate tissue inflammation and cell damages under pathological conditions, in particular, during DM (Chuah et al., 2013). However, it remains unknown whether AGEs-RAGE signaling pathway is involved in D-ribose-induced NLRP3 inflammasome activation and thereby triggers the inflammatory response in tissue or organs. The present study indeed confirmed the increased level of both AGEs and RAGE in glomeruli of mice treated with D-ribose, which could be blocked by prior treatment of AGEs formation inhibitor, AG. But whether AG could directly inhibit NLRP3 inflammasome formation needs further study. In cultured podocytes, we also found that D-ribose produced more AGEs compared to D-glucose. This led us to hypothesize that D-ribose may increase AGEs formation and thereby activate RAGE to produce NLRP3 inflammasome activation and consequent podocyte injury.

To test this hypothesis, we performed three series of cell studies to examine whether inhibition of AGEs formation, cleavage of AGE-derived protein crosslinks and gene silencing of AGEs receptor - RAGE attenuate or block D-ribose-induced NLRP3 inflammasome formation and activation. First, we demonstrated that D-ribose indeed induced NLRP3 inflammasome formation and activation in podocytes, because the inhibition of caspase-1 activity almost completely blocked D-ribose-induced NLRP3 inflammasome formation and activation. This effect of D-ribose was similar to glucose confirmed in previous studies *in vitro* (Abais et al., 2013, 2014a), indicating that a reducing sugar is a strong trigger of the NLRP3 inflammasome.

Second, we confirmed that either inhibition of AGEs formation by AG or cleavage of AGE-derived protein crosslinks by ALT substantially blocked D-ribose-induced NLRP3 inflammasome formation and activation, which was similar to the effect of caspase-1 inhibition. This suggests that increased AGEs may mediate the action of D-ribose to activate NLRP3 inflammasome in podocytes. Interestingly, L-ribose seems to have some response in cle-caspase-1 which was not inhibited by AG, but inhibited by ALT. How L-ribose works with the formation or cleavage of AGEs needs further studies. Although there is no direct evidence in literature showing the role of AGEs in mediating D-ribose-induced activation of the NLRP3 inflammasome in the kidney, some previous studies did confirm that D-ribose increased the level of AGEs in cultured human kidney 293 cells, human neuroblastoma SH-SY5Y cells, and primary cultured hippocampal neurons (Wei et al., 2009). Long-term administration of AGEs induced NLRP3 inflammasome activation and chronic renal injury (Yeh et al., 2017). In the context of AGEs activation of the NLRP3 inflammasome, there are reports that AGEs may serve as a pathogenic factor to activate NLRP3 or other inflammasome under different pathological conditions such as aging, DM, atherosclerosis (Kong et al., 2017; Deng et al., 2018). In some phagocytes such as polarized macrophages, however, AGEs significantly dampened NLRP3 inflammasome activation stimulated by influenza virus infection. It seems AGEs regulation of NLRP3 inflammasome activity depends upon tissue and cell types, which will need to be further investigated in future studies.

Finally, we tested whether RAGE is critically involved in NLRP3 inflammasome activation induced by D-ribose in podocytes. RAGE gene silencing completely blocked D-ribose-induced formation and activation of the NLRP3 inflammasome in podocytes. Based on these results, it appears that normal or increased RAGE expression is essential for D-ribose-induced NLRP3 inflammasome activation via increased AGEs. In previous studies, the role of AGEs-RAGE interaction in both diabetic nephropathy and nondiabetic renal diseases has been extensively studied and it is well accepted that AGE-RAGE signaling pathway is crucial for renal or glomerular inflammation, a hallmark of chronic kidney diseases (Abel et al., 1995; Tanji et al., 2000; Wendt et al., 2003; Che et al., 2011). For example, RAGE is expressed on normal podocytes and is upregulated in diabetic nephropathy (Tanji et al., 2000), which may drive the development of glomerular sclerosis (Wendt et al., 2003). Suppression of AGEs-RAGE pathway protected the kidney from pro-inflammatory injury, in particular, in renal glomeruli during DM (Wendt et al., 2003; Chow et al., 2006; Che et al., 2011) and antagonists of RAGE inhibited AGEs-induced NLRP3 inflammasome activation and consequent chronic renal injury (Yeh et al., 2017).

## Conclusion

In summary, the present study demonstrated that chronic administration of D-ribose induced NLRP3 inflammasome activation in podocytes and glomeruli, which was mediated by AGEs-RAGE signaling pathway. These findings elucidate a novel mechanism mediating D-ribose-induced podocyte injury and glomerular sclerosis, providing new insights into the pathogenesis of diabetic nephropathy, which may help identify D-ribose and its pathogenic action as a new therapeutic target for the treatment and prevention of diabetic nephropathy.

## Data Availability Statement

The data used to support the findings of this study are available from the corresponding author upon request.

## Ethics Statement

The studies involving animal study was reviewed and approved by the Institutional Animal Care and Use Committee of the Virginia Commonwealth University.

## Author Contributions

P-LL, JR, and WL contributed to the conception of the study. JH, GL, and QZ contributed significantly to analysis and manuscript preparation. JH and GL performed the data analyses and wrote the manuscript. JH, GL, and P-LL helped perform the analysis with constructive discussions.

## Conflict of Interest

The authors declare that the research was conducted in the absence of any commercial or financial relationships that could be construed as a potential conflict of interest.
